# The Effects of Multi-Walled Carbon Nanotubes and Steel Fibers on the AC Impedance and Electromagnetic Shielding Effectiveness of High-Performance, Fiber-Reinforced Cementitious Composites

**DOI:** 10.3390/ma12213591

**Published:** 2019-10-31

**Authors:** Namkon Lee, Sungwook Kim, Gijoon Park

**Affiliations:** Structural Engineering Research Division, Korea Institute of Civil Engineering and Building Technology, 283 Goyangdae-Ro, Ilsanseo-Gu, Goyang-Si, Gyeonggi-Do 10223, Korea; swkim@kict.re.kr (S.K.); joon7767@kict.re.kr (G.P.)

**Keywords:** MWCNTs, AC impedance, electromagnetic shielding, HPFRCC, steel fiber

## Abstract

This study aimed to investigate the effect of multi-walled carbon nanotubes (MWCNTs) and steel fibers on the AC impedance and electromagnetic shielding effectiveness (SE) of a high-performance, fiber-reinforced cementitious composite (HPFRCC). The electrical conductivity of the 100 MPa HPFRCC with 0.30% MWCNT was 0.093 S/cm and that of the 180 MPa HPFRCC with 0.4% MWCNT and 2.0% steel fiber was 0.10 S/cm. At 2.0% steel fiber and 0.3% MWCNT contents, the electromagnetic SE values of the HPFRCC were 45.8 dB (horizontal) and 42.1 dB (vertical), which are slightly higher than that (37.9 dB (horizontal)) of 2.0% steel fiber content and that (39.2 dB (horizontal)) of 0.3% MWCNT content. The incorporation of steel fibers did not result in any electrical percolation path in the HPFRCC at the micro level; therefore, a high electrical conductivity could not be achieved. At the macro level, the proper dispersion of the steel fibers into the HPFRCC helped reflect and absorb the electromagnetic waves, increasing the electromagnetic SE. The incorporation of steel fibers helped improve the electromagnetic SE regardless of the formation of percolation paths, whereas the incorporation of MWCNTs helped improve the electromagnetic SE only when percolation paths were formed in the cement matrix.

## 1. Introduction

A high-performance, fiber-reinforced cementitious composite (HPFRCC) has a compressive strength of more than 150 MPa [1]. It mainly consists of cement, filler, aggregate, microsilica, and superplasticizer. Fibers, such as steel fibers, PE fibers, or PVA fibers, can be added to increase its tensile strength. An HPFRCC has higher strength and durability than normal-strength concrete and high-strength concrete, owing to its low water-to-binder ratio and high amount of cementitious material [2].

Among the constituent materials of HPFRCCs, steel fibers have a high tensile strength of 2500 MPa and a good electrical conductivity. The high electrical conductivity of steel fibers can make the HPFRCC conductive. The conductivity of normal-strength concrete, particularly when containing steel fibers and carbon nanotubes (CNTs) [3,4,5,6,7,8,9], has been studied. However, studies on the electrical conductivity of HPFRCCs are lacking.

The electrical conductivity of concrete containing steel fibers and CNTs can be measured using alternative current impedance spectroscopy (AC-IS) to investigate dispersion problems such as the alignment and agglomeration of fibers. AC-IS is a promising technique to reveal the variations in the microstructure during the hydration of cement pastes. It is a non-destructive technique that can be used to electrically evaluate the fiber orientation in fiber-reinforced cement-matrix composites. An electrical method based on the AC-IS offers a convenient, non-destructive, and quantitative method for quality assurance in the production of fiber-reinforced cement composites [10]. Studies [3,11,12,13] have shown that the AC-IS can be utilized to investigate the microstructure of cementitious composites with conductive carbon fibers or steel fibers.

Because of the complicated microstructure of cement-based materials, simplified physical models have been proposed, based on which equivalent circuit model has been established to represent the electrical properties of concrete [13]. In the electrical circuit model [14], only three conductive paths, the continuous conductive paths, discontinuous conductive paths, and non-conductive paths, were considered. Based on this model, the effects of hydration time, silica fume replacement, and water-to-cement ratio on the high-frequency arc were determined [15]. In addition, the microstructure of the interfacial transition zone (ITZ) between the different aggregates and the cement paste was studied [16]. The relationships between the impedance characteristics and the pore solution concentration, porosity, and mean pore size have been established to reflect the degree of hydration and microstructural evolution of cement-based materials [17,18].

Fiber dispersion issues in macro and micro-composites, including orientation, coarse-scale segregation, and local aggregation, can be investigated using the AC-IS [3]. Moreover, the fiber loading in cement-based composites with non-percolating fibers can be predicted in terms of the fiber volume fraction and fiber aspect ratio (length/diameter) [11]. Experimental results have shown that the AC-IS can potentially serve as a reliable, convenient, and non-destructive tool to evaluate fiber dispersion issues (such as fiber orientation, global segregation, and local aggregation) in cement-based composites with short conductive fibers [10].

Wansom et al. (2006) [3] investigated the impedance response of fiber-reinforced cementitious composites with multi-walled carbon nanotubes (MWCNTs). Three impedance arcs were observed in the Nyquist plots (imaginary impedance versus +real impedance). Three important parameters, namely Rcusp (at high frequency), *R*_DC_ (DC resistance of reinforced-cement matrix measured using the four-point method), and *R*_DC_ (DC resistance of unreinforced-cement matrix), were identified in the study. The intersection of the electrode arc and the intermediate frequency (*R*_DC_ for FRCs) corresponds to the DC resistance of the composite, and the intersection of the two bulk arcs (*R*_cusp_) corresponds to the impedance of the composite. A reduction in *R*_DC_ (FRC) from the matrix resistance is indicative of a nanotube percolating network, and a reduction in *R*_cusp_ is indicative of a discontinuous fiber–fiber path. Therefore, AC-IS measurements can help discriminate percolation and discontinuous fiber effects in CNT-based cementitious composites, with the potential for characterizing dispersion issues (e.g., clumping/aggregation) in cementitious composites [3].

Adding steel fibers to cement does not significantly affect the DC and AC matrix resistivity of the cement mortar. However, steel fibers lead to a drastic reduction in the frequency associated with the AC matrix resistivity, ranging from ~1 kHz in plain mortar to ~1 Hz in steel fiber-reinforced mortar. These findings reveal the need to appropriately adjust the frequency while measuring the AC resistivity of steel fiber-reinforced cementitious materials [4].

The AC-IS method has been mostly used to investigate the microstructural characteristics of cement matrix and fiber dispersion issues in cement/fiber composites. Steel fibers and CNTs are recommended for electromagnetic shielding, as they are conductive; their use as macrofibers and microfibers, respectively, is expected to create a synergistic effect on the electrical conductivity of concrete by providing a conductive network in the cement matrix, giving rise to a percolation threshold. Studies on the electrical conductivity and electromagnetic shielding effectiveness (SE) of steel fiber/CNT-incorporated cementitious composites are lacking [19,20,21]. In particular, there is no study on the electromagnetic shielding effectiveness of HPFRCCs. Steel fibers are generally utilized to increase the ductility of HPFRCCs and can potentially provide a good electromagnetic shielding owing to their conductive property. CNTs can be better dispersed in an HPFRCC matrix than in an ordinary Portland cement (OPC) matrix, as HPFRCCs have an extremely low water-to-cement ratio and contain a high amount of silica fume, which plays a role of a ball bearing effect. Under oven drying, a low water-to-cement ratio can help improve the dispersion level of CNTs in the cement paste [22,23].

This study aimed to investigate the electrical conductivity and electromagnetic shielding effectiveness of HPFRCCs. To improve the electrical properties of HPFRCCs, MWCNTs and steel fibers were added to the HPFRCC mixes. The electrical conductivity was measured using the AC-IS method, and the electromagnetic shielding effectiveness was measured inside a dual shielding room in accordance with MIL-STD-188-125.

## 2. Materials and Methods

### 2.1. Materials

The ordinary Portland cement (OPC, ASTM C150 Type I) used in this study was provided by Sungshin Cement Corp., South Korea. The OPC cement had a Blaine fineness of 3700 cm^2^/g and a specific gravity of 3.17. Silica fume was supplied by Elkem Corp., South Korea. Silica powder (average grain size: 14 μm) and quartz sand with a diameter in the range of 100–800 μm were employed for the fabrication of HPFRCCs. Steel fibers (diameter: 0.2 mm, length: 19.5 mm) and MWCNTs (diameter: 6–9 nm, length: 50–200 μm, purity: min 98.5%) were used as conductive materials. In addition, an MWCNT liquid solution prepared by dispersing MWCNTs in distilled water by sonication was used as a conductive material.

Table 1 lists the chemical composition of the OPC and fly ash used in this study, including the mineralogical composition of the anhydrous OPC determined by XRD Rietveld analysis. The concentration of the MWCNT solution was 2%. Table 2 lists the physical properties of the steel fibers, wherein $l_{f}$ and $d_{f}$ are, respectively, the length and diameter of the steel fibers.

### 2.2. Mixture Proportions and Sample Preparation

Table 2 lists the mixture proportions of the HPFRCC. The mix proportions are divided into two categories: HPFRCCs with compressive strengths of 100 and 180 MPa. The following notations are used to identify the samples throughout this paper. Table 3 gives the designations of the HPFRCC mixes with their respective mix compositions, where H represents the plain 100 MPa HPFRCC; UH represents the plain 180 MPa HPFRCC; N and LN represent the HPFRCCs with the MWCNT powder and MWCNT liquid solution, respectively; and the following figure is the MWCNT content (%) by mass of cement; S following H indicates the incorporation of steel fibers in the HPFRCC. For example, UHS_LN0.3 represents the 180 MPa HPFRCC containing steel fibers and MWCNT liquid solution where the MWCNT content is 0.3% by mass of cement.

The water-to-binder (cement + microsilica) ratio was 0.30 for the 100 MPa HPFRCC and the ratios were 0.2, 0.25, and 0.30 for the 180 MPa HPFRCC. The amount of superplasticizer agent was adjusted to meet the mini slump flow requirement of 200 mm (measured after 25 times hits); however, some samples could not meet this requirement because of the high amount of MWCNTs added.

Steel fibers were added to the HPFRCC at 0, 0.1, 0.5, 1.0, 2.0, and 3.0 vol%. The MWCNTs were added to the HPFRCC in the range of 0–0.5% by weight of cement. 

The HPFRCC was manufactured in accordance with the study conducted by Lee et al. (2018) [24]. The fresh HPFRCC was placed in 60 mm × 60 mm × 160 mm molds for AC-IS measurements. For the electromagnetic shielding effectiveness test, the HPFRCCs were casted into 300 mm × 300 mm × 100 mm molds. The molds were immediately covered with poly-plastic vinyl sheets to prevent surface drying.

The HPFRCC samples were cured at a temperature of 20 °C and RH > 99% in sealed conditions for the initial 24 h. The samples were then cured in a water bath at a temperature of 90 °C for 72 h. Finally, the samples were oven-dried at 60 °C for 72 h to prevent the pore solution effect on the electrical conductivity.

### 2.3. Testing Methods

The diffraction pattern of the powdered sample was obtained using a conventional X-ray diffractometer (SmartLab, Rigaku), with CuKα radiation at 45 kV and 200 mA, a step size of 0.01°, and 0.2 s per step over a 2θ range of 5–70°. The resulting pattern was analyzed with the inorganic crystal structure database (ICSD) using X’pert HighScore Plus software. To quantify the mineralogical composition, a Rietveld analysis was conducted by refining the scale factors, peak asymmetry, zero shift, specimen displacement, and unit cell parameters with a manually fixed background. 

An unconfined compressive strength test was conducted using a 300 kN universal testing machine in accordance with ASTM C39, and the strength was averaged from three samples.

The AC impedance of the 60 × 60 × 160 mm specimen embedded with two copper electrodes (20 × 60 × 0.5 mm) was measured at an interval of 30 mm. LCR meters (Keysight Technologies, model: E4980A) were employed for the AC-impedance measurement. The frequency was swept from 1 MHz down to 1 Hz using a logarithmic point spacing of 50 points. To minimize the conduction effect of the pore solution, the tests were performed after the sample was dried at 60 °C for three days. To confirm the true bulk resistance of the HPFRCC specimens, four-point DC measurements were also conducted.

**Figure 1:**
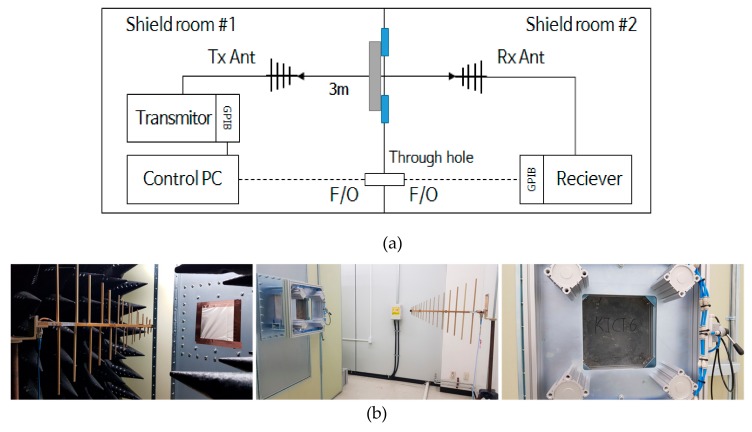
Shielding effectiveness measurement system. (**a**) System configuration, (**b**) Tx (Transmission) antenna, Rx (Receiving) antenna, and sample installation.

The shielding effectiveness measurement of the HPFRCC samples was conducted in accordance with the military standard MIL-STD-188-125. Figure 1 shows the system diagram used for measuring the shielding effectiveness of the HPFRCC samples. The measurement location was isolated by a metal wall between the two shield rooms. To minimize the electromagnetic waves flowing into the receiver, the receiving antenna and the receiver were placed in shield room #2, and the other electronic equipment was installed in shield room #1 where the transmitting antenna was located. The two antennae used for transmission and reception were log-periodic antennae with a measurement bandwidth of 290 MHz to 2 GHz. The antennae were placed in the line of sight through the aperture of the jig on the metal wall. The distance between the antennae was 3 m, and the height was 1.2 m, which was the same as the center of the aperture.

The pore size distribution of the HPFRCC was measured using mercury intrusion porosimetry (MIP) in accordance with ASTM D4284-07 on an AutoPore IV machine (Micromeritics Corp). The surface tension and contact angle of mercury were assumed to be 485 dynes/cm and 130°, respectively. The 5 mm cubic samples were prepared by cutting the 50 mm cubic samples using a diamond cutting machine (PRESI, Model T202) for the MIP test.

## 3. Results and Discussion

### 3.1. AC Impedance Spectrum Response

**Figure 2:**
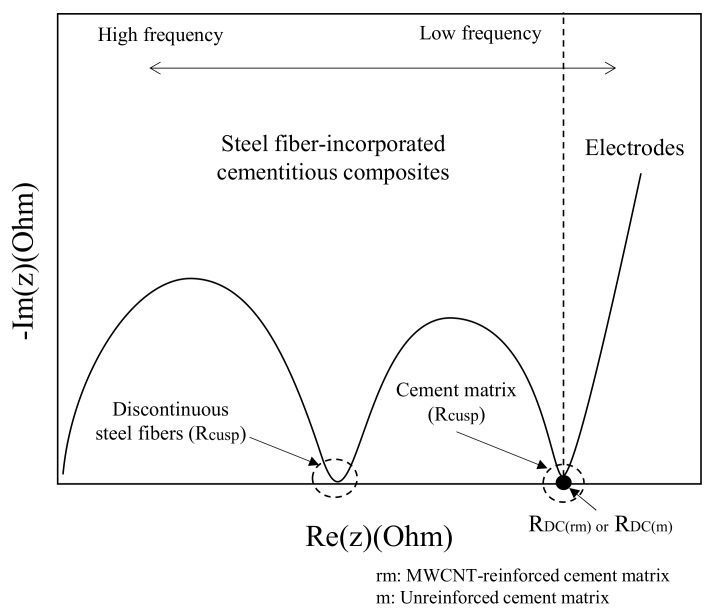
Typical Nyquist plots for steel fiber-incorporated cementitious composites.

As mentioned by Wansom et al. (2006) [3], the typical impedance responses of the HPFRCC with multi-walled carbon nanotubes and steel fibers are plotted, as shown in Figure 2. Two impedance arcs can be observed in the Nyquist plots, which were obtained from the AC impedance results in accordance with [25]. The Nyquist plot obtained from the AC-IS measurement results can help differentiate the impedances between the steel fiber/cement composites and the cement matrix. Three parameters, namely the left-side *R*_cusp_ (at high frequency), right-side *R*_cusp_ (at low frequency), and *R*_DC_ (DC resistance of unreinforced or reinforced-cement matrix measured using the four-point method), are identified in Figure 2. The left-side *R*_cusp_ was attributed to the short-circuit current of the steel fibers in the HPFRCC, and the right-side *R*_cusp_ was attributed to the AC resistance of the cement matrix in the HPFRCC

**Figure 3:**
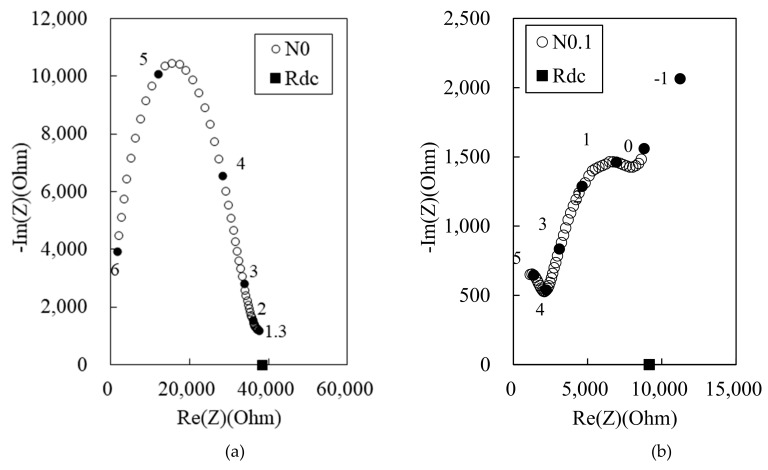

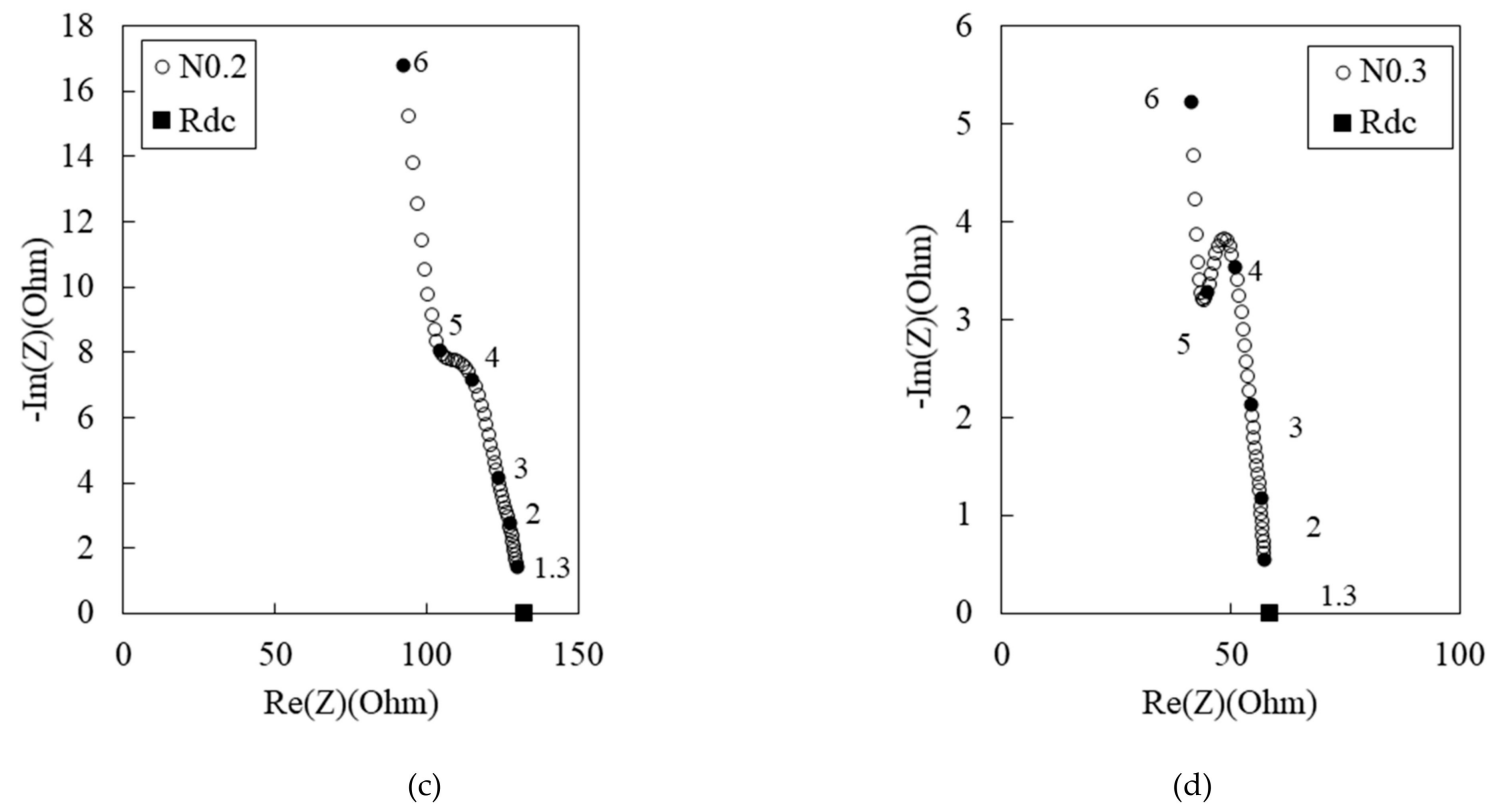
Experimental Nyquist plots for 100 MPa HPFRCC with (**a**) 0%, (**b**) 0.1%, (**c**) 0.2%, and (**d**) 0.3% multi-walled carbon nanotubes (MWCNTs) (length: 100–200 μm, diameter: 6–9 nm) (Frequency markers are shown as darkened points with the numbers corresponding to the log of frequencies in Hz).

Figure 3 shows the experimental Nyquist plots for the 100 MPa HPFRCC with varying MWCNT contents: (a) 0%, (b) 0.1%, (c) 0.2%, and (d) 0.3%. The 100 MPa HPCC with 0.4% MWCNT could not be produced, because the addition of 0.4% MWCNT led to poor flowability of the HPFRCC. The N0 sample exhibits a single-arc behavior, and *R*_m_ (real impedance at the right-side cusp point between the single bulk arc and the electrode arc) is 38350 Ω. At 0.1% MWCNT, the dual arcs are more prominent, and *R*_cusp_ and *R*_rm_ decrease to 2233 and 8035 Ω, respectively. At 0.2% MWCNT, the dual-arc behavior is not clear; nevertheless, one of the dual arcs appears as a weak shoulder. As shown in Figure 3b–d, the dual arcs are observed because of the addition of MWCNTs despite the absence of steel fibers; *R*_cusp_ and *R*_rm_ both decrease with the increase in the MWCNT content.

**Figure 4:**
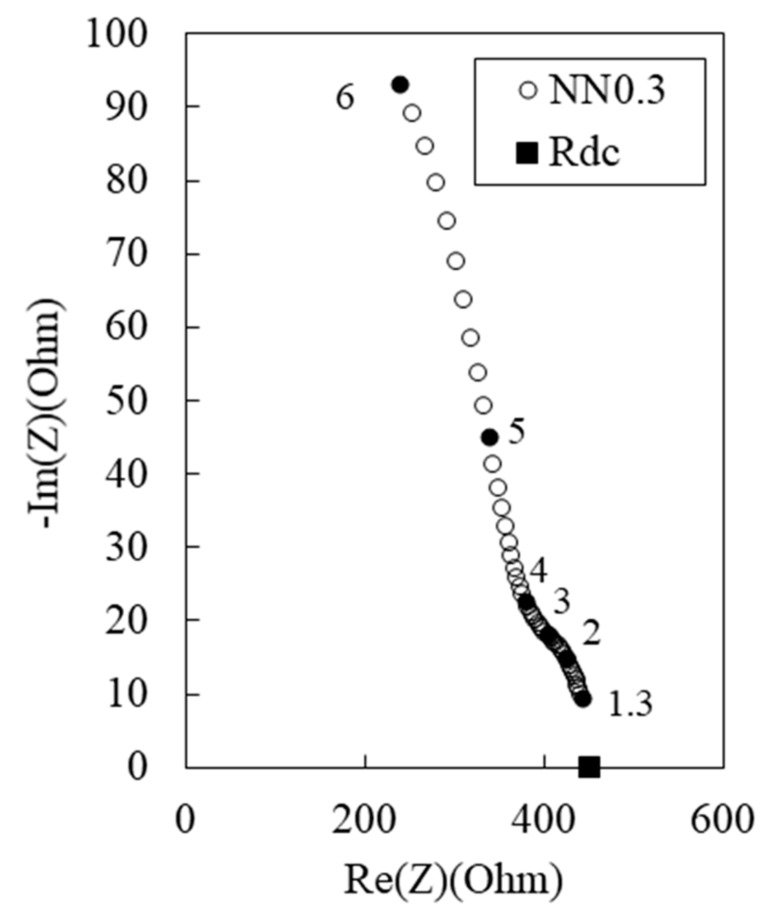
Experimental Nyquist plot for 100 MPa-HPCC with 0.3 wt.% MWCNT (length: 50–150 μm, diameter: 6–9 nm).

Figure 4 shows the Nyquist plot for the 100 MPa HPCC with 0.3% MWCNT of a shorter length. At the same MWCNT content, the use of MWCNTs with a longer length (Figure 3d) leads to a decrease in *R*_cusp_ and *R*_rm_. This suggests that the longer length MWCNTs are beneficial for the formation of a percolation path.

**Figure 5:**
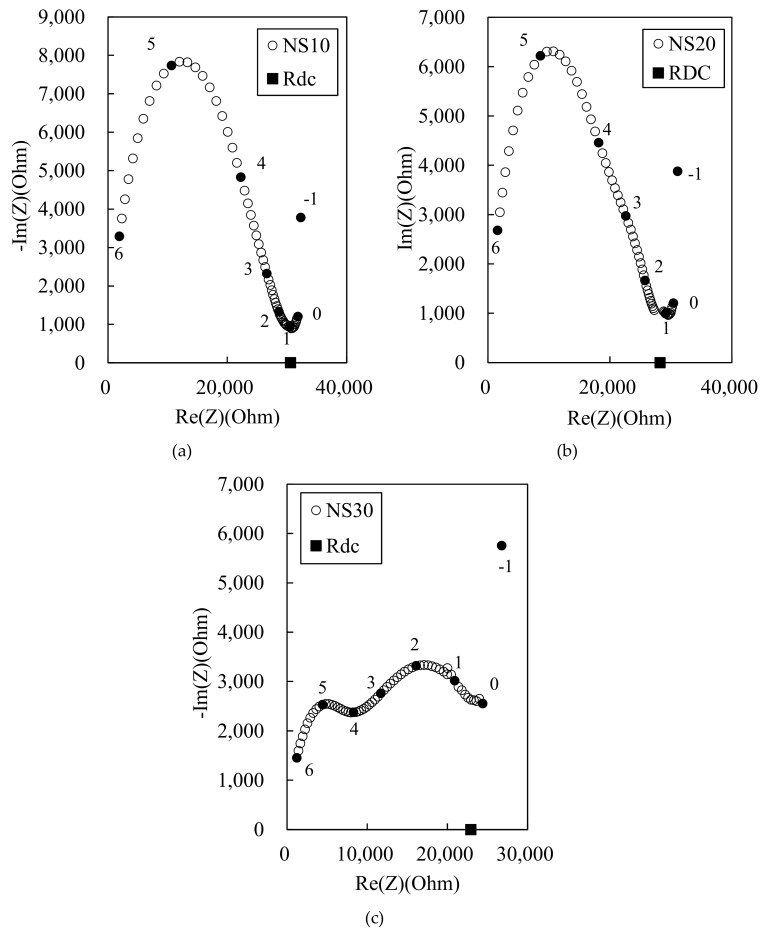
Experimental Nyquist plot for 100 MPa-HPCC with MWCNT solution at (**a**) 10%, (**b**) 20%, and (**c**) 30% replacement of mix water.

Figure 5 shows the Nyquist plots for the 100 MPa HPFRCC, where 10%, 20%, and 30% of the mix water are replaced by the MWCNT liquid solution. The effect of MWCNT liquid solution on the impedance is little compared with that of the powder MWCNT. Only the SN30 sample shows visible dual-arc behavior, whereas no distinct two cusp points can be observed in the SN10 and SN20 samples. This is probably due to the poor dispersion of the MWCNTs in the cement matrix when using the MWCNT liquid solution. Another reason is that the higher amount of surfactant in the MWCNT liquid solution can lead to the formation of air voids during the manufacturing process, disturbing the percolation path. The increase in the air voids can be confirmed from the MIP results in Section 3.4.

**Figure 6:**
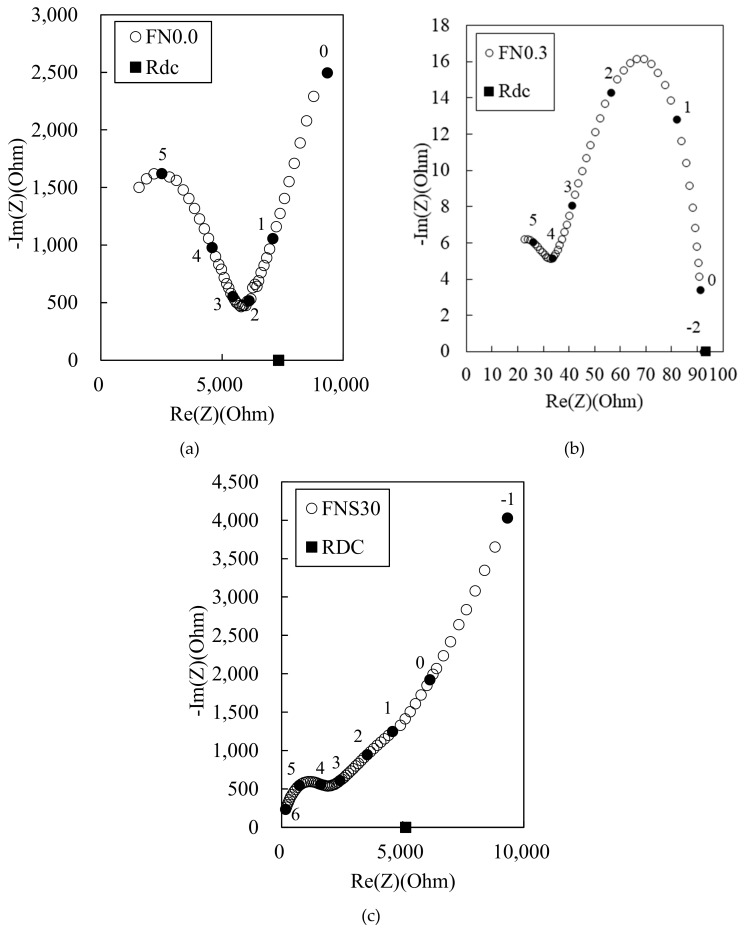
Experimental Nyquist plot for 100 MPa-HPFRCC with (**a**) 0% and (**b**) 0.3% MWCNT, and with (**c**) MWCNT solution at 30% replacement of mix water.

Figure 6 shows the Nyquist plots for the 100 MPa HPFRCC containing steel fibers. In Figure 6a, *R*_cusp_ at high frequency can be observed, but it is difficult to distinguish *R*_cusp_ at low frequencies and the electrode arc in the HPFRCC with steel fibers. This behavior has been previously reported [3]. Nevertheless, given that *R*_cusp_ at low frequency (corresponding to the impedance of the reinforced composite) coincides with *R*_DC(rm)_ (the four-point DC resistance of the reinforced composite) in conductive fiber/cement composites [3,4,10], we can find the positions of *R*_cusp_ at high frequency, *R*_cusp_ at low frequency, and *R*_DC_ from the Nyquist plots of the HPFRCC. At 0.3% MWCNT and 2.0% steel fiber (Figure 6b), the dual arcs can be observed, and *R*_cusp_ at high frequency and *R*_rm_ are significantly decreased, compared with those of HPFRCCs with 0% MWCNT and 2.0% steel fiber. 

This result supports the synergy effect of the steel fibers and MWCNTs for the formation of an electrical percolation path. The use of the MWCNT liquid solution had a much lesser effect on the decrease in the impedance of HPFRCC (increase in electrical conductivity).

**Figure 7:**
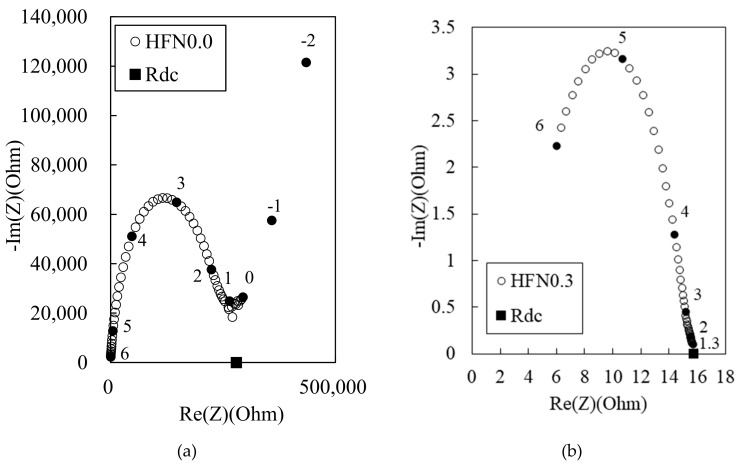
Experimental Nyquist plot for 180 MPa-HPFRCC with (**a**) 0% and (**b**) 0.3% (w/c ratio of 0.25).

Figure 7 shows the Nyquist plots for the 180 MPa HPFRCC. *R*_m_ is much higher in the 180 MPa HPFRCC than in the 100 MPa HPFRCC (Figure 6a). This is probably because the fly ash in the 100 MPa HPFRCC contains 5.42% Fe_2_O_3_, which serves as conductive particulate matter, or because the 180 MPa HPFRCC contains a higher amount of air voids, which play a role as an insulating material, resulting from the use of a higher amount of SP agent. The addition of 0.3% MWCNT in the 180 MPa HPFRCC resulted in the lowest *R*_cusp_ at a high frequency and the lowest *R*_rm_ among the samples.

**Figure 8:**
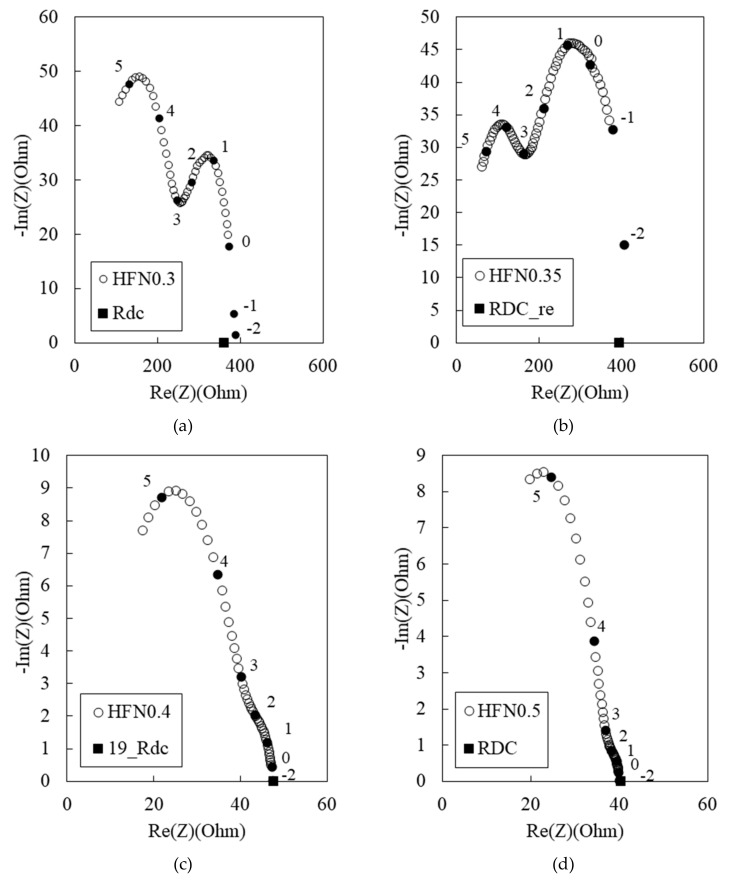
Experimental Nyquist plots for 180 MPa-HPFRCC (w/c ratio of 0.3) with (**a**) 0.3%, (**b**) 0.35%, (**c**) 0.4%, and (**d**) 0.5% MWCNT.

Figure 8 shows the Nyquist plots for the 180 MPa HPFRCC with 0.3%, 0.35%, 0.4%, and 0.5 MWCNTs. *R*_cusp_ at high frequency and *R*_rm_ hardly decreased with the increase in the MWCNT content from 0.3% to 0.35%. They significantly decreased when the MWCNT content was increased from 0.35 to 0.40%. This indicates that the electrical percolation threshold of the 180 MPa HPFRCC containing 2.0% steel fiber is between 0.35% and 0.40% MWCNT content.

**Figure 9:**
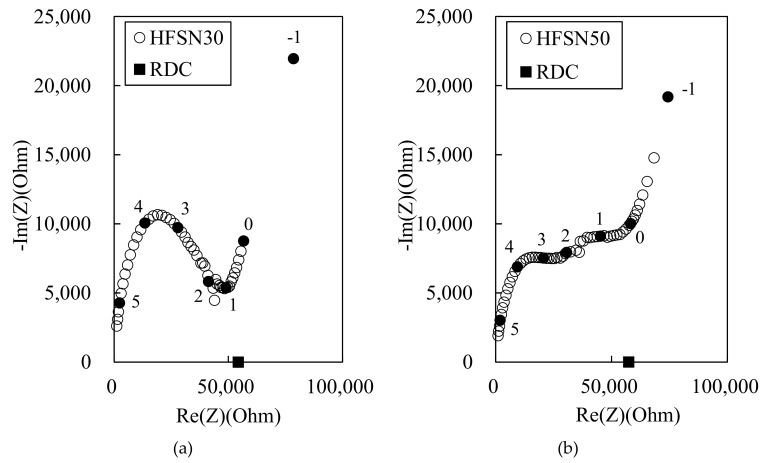
Experimental Nyquist plot for 180 MPa-HPFRCC with MWCNT solution at (**a**) 30% and (**b**) 50% replacement of mix water.

Figure 9 shows the Nyquist plots for the 180 MPa HPFRCC with the MWCNT liquid solution. The MWCNT liquid solution has no effect in decreasing *R*_cusp_ and *R*_rm_ similar to the case of the 100 MPa HPFRCC. 

**Figure 10:**
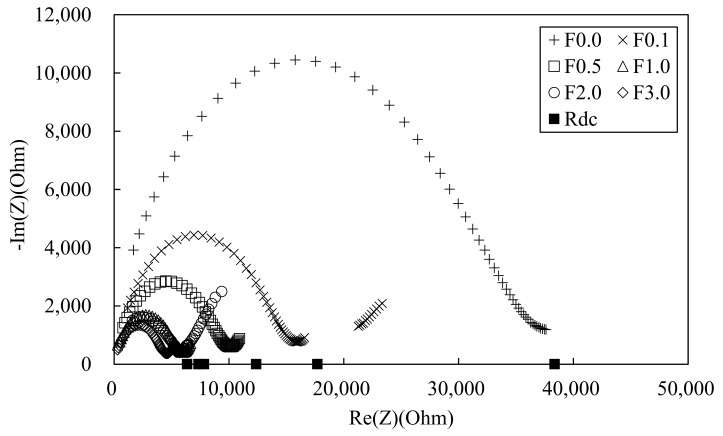
Experimental Nyquist plot for 100 MPa-HPFRCC with (a) 0.1%, (b) 0.5%, (c) 1.0%, (d) 2.0%, and (e) 3.0% steel fiber (volume %).

Figure 10 shows the Nyquist plots for the 100 MPa HPFRCC containing 0, 0.1, 0.5, 1.0, 2.0, and 3.0 wt.% steel fiber. Although steel fibers were added to the HPFRCC, only Rcusp at high frequency, among the two Rcusp points (Figure 2), can be clearly observed. Another Rcusp (at low frequency), which is assigned to the impedance of the cement matrix, is not obvious and overlaps with the electrode arc [5]. This is probably because of the similar time constants between the oxide films on the steel fibers and those on the copper measurement electrodes [5]. Rcusp at low frequency, the impedance of the cement matrix in the HPFRCC, can be alternatively distinguished from the electrode arc by obtaining Rdc through the four-point DC measurements.

### 3.2. Compressive Strength and Flowability

**Figure 11:**
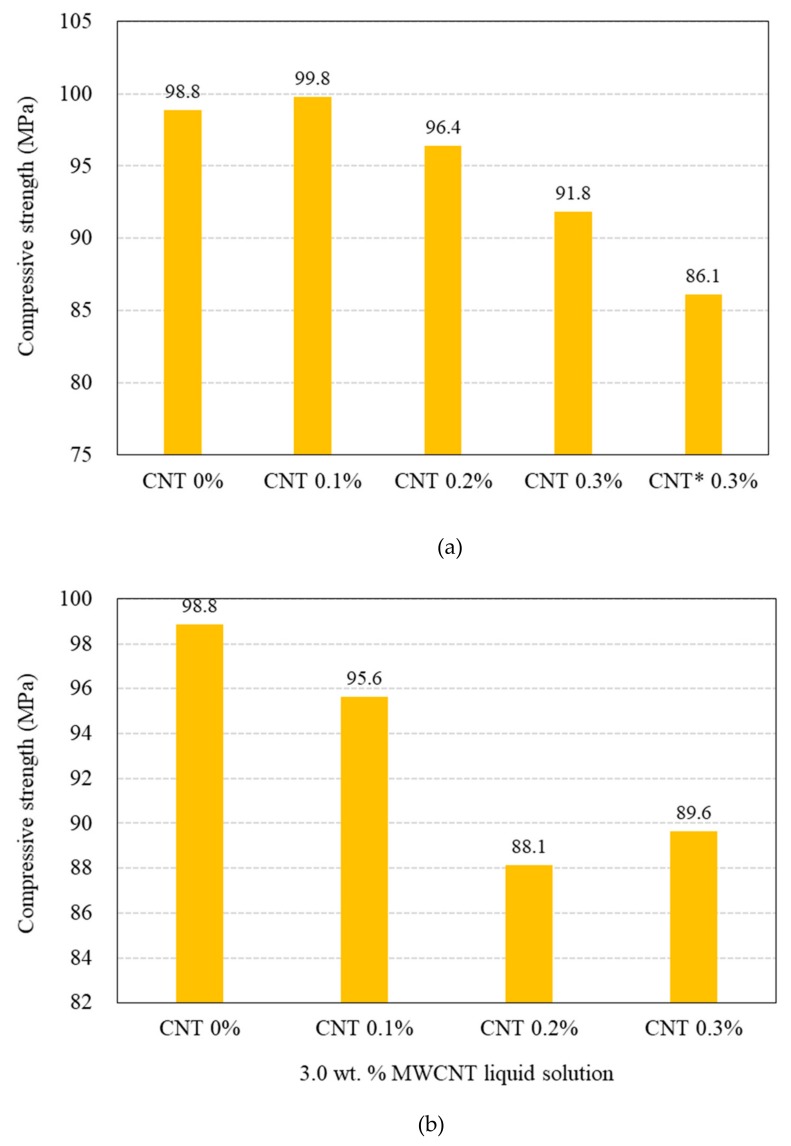

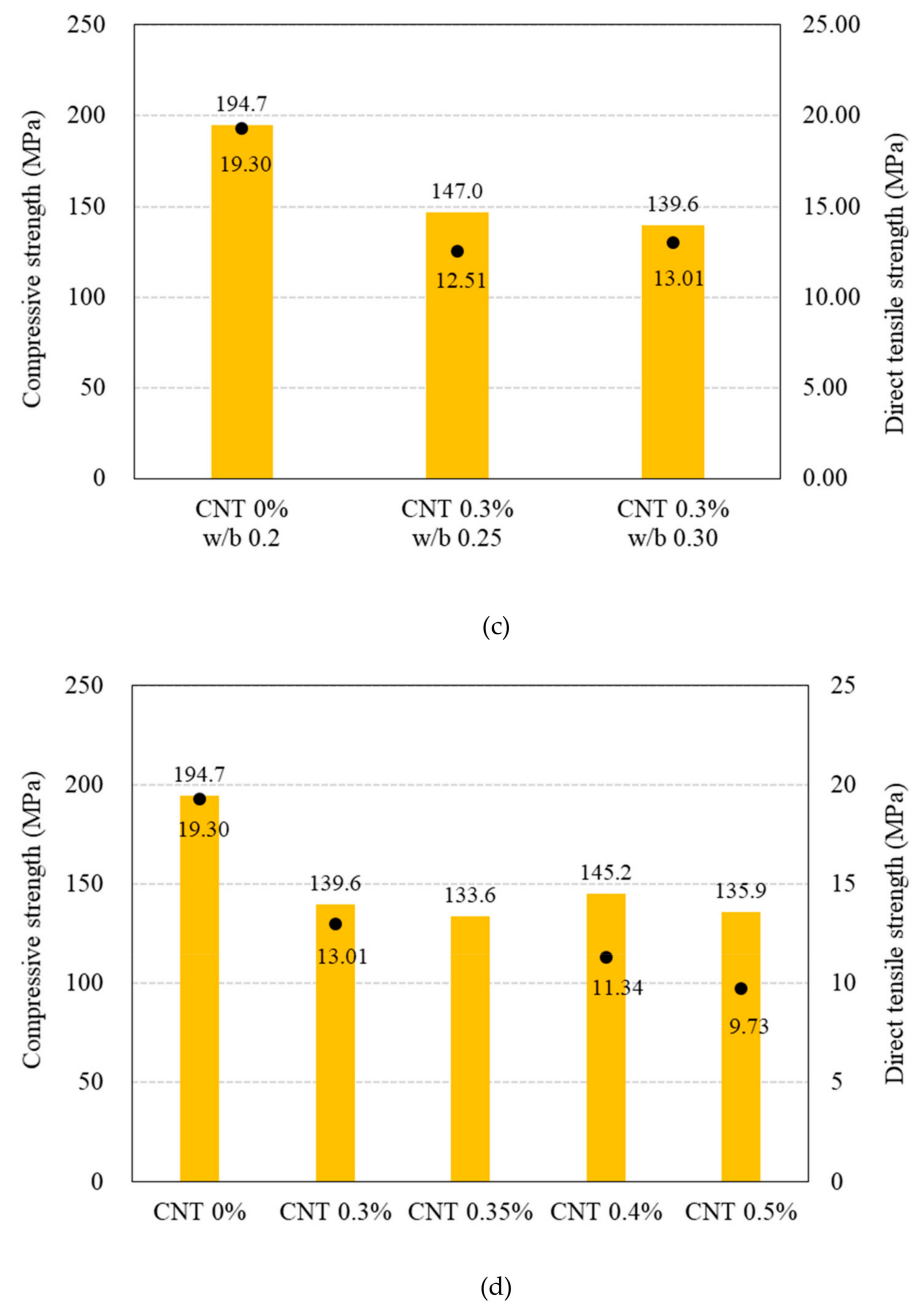

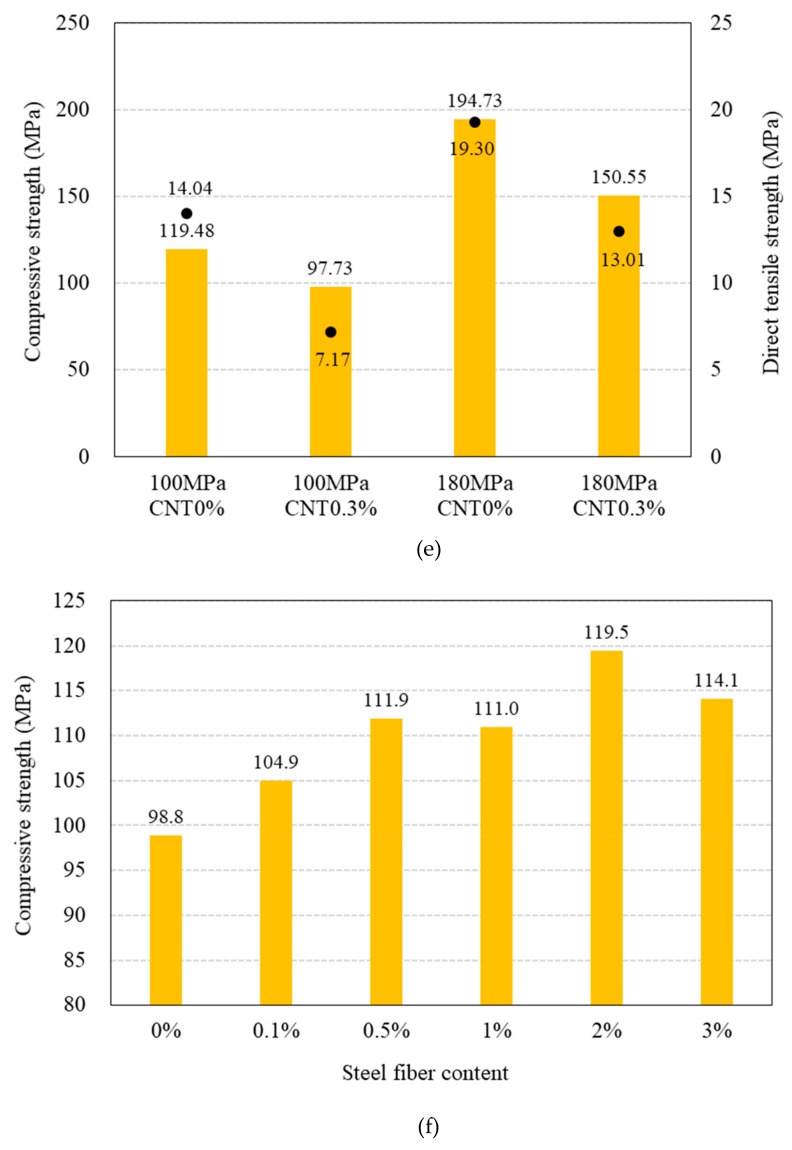
Compressive strength and direct tensile strength results for 100 MPa and 180 MPa-HPFRCC w/wo steel fibers. (**a**) Use of powder-type MWCNT in 100 MPa-HPFRCC without steel fiber; (**b**) use of liquid-type MWCNT solution in 100 MPa-HPFRCC without steel fiber; (**c**) use of powder-type MWCNT in 180 MPa-HPFRCC with 2.0% steel fiber; (**d**) use of powder-type MWCNT in 180 MPa-HPFRCC with 2.0% steel fiber; (**e**) use of liquid-type MWCNT in HPFRCC with 2.0% steel fiber; (**f**) steel fiber contents in 100 MPa-HPFRCC. * MWCNT used in 0.3% CNT* sample had a length in the range of 50–150 μm, which is shorter than that (100–200 μm) of MWCNTs used in the other samples.

Figure 11 shows the compressive and direct tensile strengths of the 100 and 180 MPa HPFRCCs with and without steel fibers. As shown in Figure 11a, the compressive strength of the 100 MPa HPFRCC without the steel fibers at 0.1% MWCNT is slightly greater than that at 0% MWCNT; it then decreases with increasing MWCNT content (%). The compressive strength of the sample (CNT* 0.3%) with shorter length MWCNTs (50–150 μm) is remarkably lower than that of the 0.3% CNT sample. 

The use of the liquid-type MWCNT solution resulted in a greater decrease in the compressive strength of the 100 MPa HPFRCC without the steel fibers (Figure 11b). At a MWCNT content of 0.1%, the compressive strengths of the powder-type MWCNT sample and the liquid-type MWCNT sample are 99.8 and 95.6 MPa, respectively; at a MWCNT content of 0.3%, the values are 91.8 and 89.6 MPa. 

Figure 11c shows the effect of w/b ratio on the compressive strength of the 180 MPa HPFRCC with 2.0% steel fiber and MWCNT. To disperse the MWCNT properly in the 180 MPa HPFRCC and obtain the targeted flowability (200 mm), the w/b ratio needs to be increased. As the w/b ratio is increased from 0.20 to 0.25, the compressive strength decreases from 194.7 to 147 MPa and the direct tensile strength decreases from 19.30 to 12.51 MPa. Figure 11d shows the effect of MWCNTs on the compressive strength of the 180 MPa HPFRCC. Up to a MWCNT content of 0.3%, the strength significantly reduces from 194.7 to 139.6 MPa. As the MWCNT content is increased from 0.3% to 0.5%, the compressive strength hardly decreases, whereas the direct tensile strength decreases from 13.01 to 9.73 MPa.

Figure 11f shows the effect of steel fibers on the compressive strength of the 100 MPa HPFRCC. With the increase in the steel fiber content, the compressive strength increases except for the 3.0 vol.% sample, which exhibits an excessive clumping problem because of the steel fibers. 

Table 4 lists the mini slump flow results. Up to an MWCNT content of 0.1% (N0.1 sample), the slump flow does not decrease; it decreases to 150 mm when the MWCNT content is 0.3% (N0.3 sample). In the case of the liquid-type MWCNT solution, the slump flow is as high as 220 mm at an MWCNT content of 0.2%. With the addition of steel fibers, the slump flow is 130 mm at an MWCNT of 0.3% (H100N sample). When the w/b ratios are 0.25 and 0.30, the slump flow values are 150 and 230 mm, respectively. When the MWCNT contents are 0.3%, 0.35%, 0.4%, and 0.5%, the slump flow decreases to 230, 170, 160, and 150 mm, respectively. The mini slump flow of the HPFRCC is maintained as high as 200 mm when the MWCNT amount is in the range of 0.30%–0.35%.

### 3.3. Electrical Conductivity and Percolation Path/Threshold

**Figure 12:**
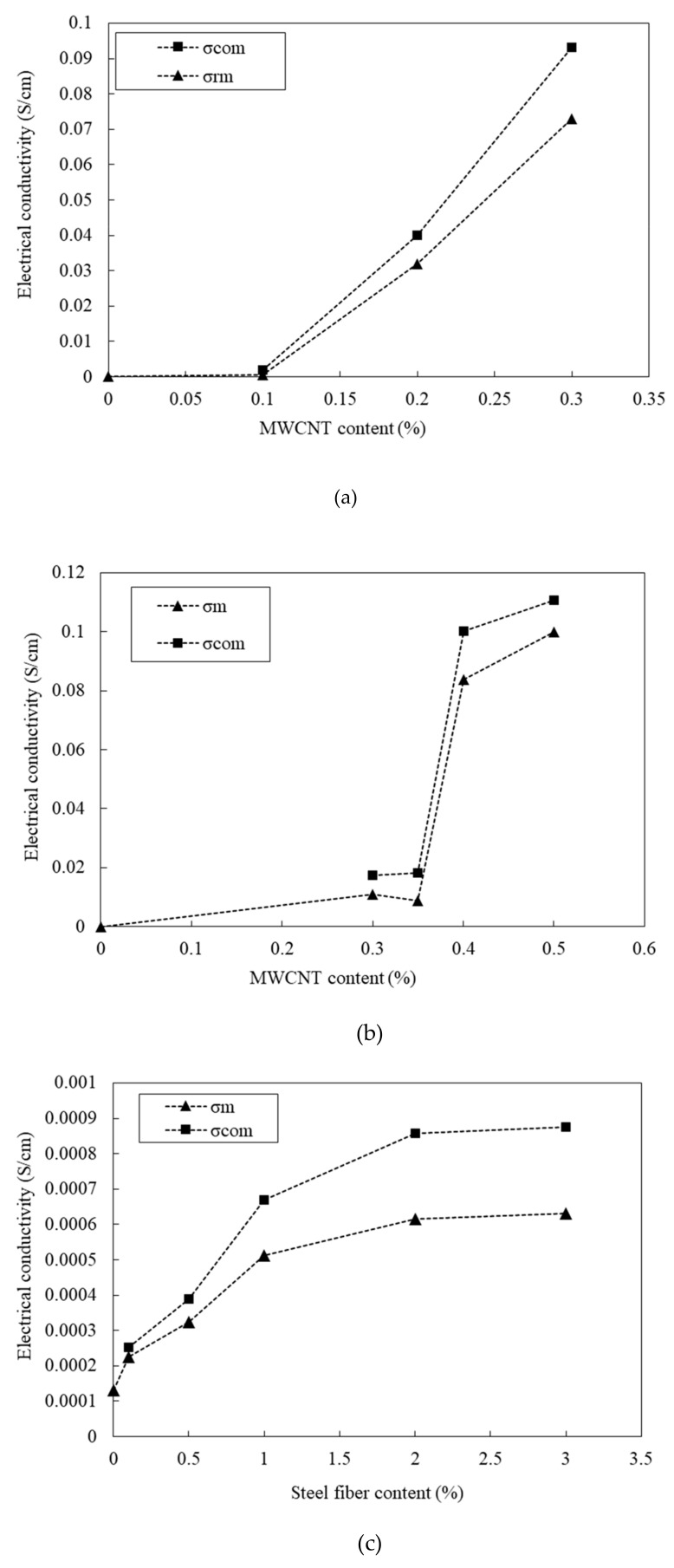
Electrical conductivity of 100 MPa and 180 MPa-HPFRCC w/wo MWCNT and/or steel fiber. (**a**) 100 MPa-HPFRCC without steel fiber; (**b**) 180 MPa-HPFRCC with steel fiber; (**c**) 100 MPa-HPFRCC with steel fiber.

Figure 12 shows the electrical conductivity of the 100 and 180 MPa HPFRCCs. The electrical conductivity (σ) can be calculated as follows:

(1) $$\sigma \;=\frac{1}{R}\times \frac{L\;}{A},$$

where, *R* is the resistance (Ω); *A* is the area of contact between the material and the electrodes (cm^2^); *L* is the distance between the electrodes (cm); and σ is the electrical conductivity (S/cm). In Figure 12, σ_(com)_, σ_(mat)_, and σ_(r-mat)_ denote the electrical conductivities of the composites (matrix + steel fiber), unreinforced matrix, and MWCNT-reinforced matrix (matrix + MWCNT), respectively.

Figure 12a shows the effect of MWCNT content on the electrical conductivity. As it was impossible to prepare the 100 MPa HPFRCC with 0.4% MWCNT because of the low flowability; the MWCNT content was limited to 0.3% in the 100 MPa HPFRCC. The electrical conductivity tends to increase with the amount of MWCNTs. At an MWCNT content of 0.1%, the electrical conductivity (σ_com_) of the HPFRCC at the left-side cusp point (Refer to Figure 2) is quite low (0.002 S/cm) and that (σ_rm_) of the MWCNT-reinforced matrix of the HPFRCC is 0.0005 S/cm. At an MWCNT content of 0.2%, the electrical conductivity (σ_com_) of the 100 MPa HPFRCC is significantly increased to 0.04 S/cm. At an MWCNT content of 0.3%, the value (σ_com_) reaches 0.093 S/cm. The 180 MPa HPFRCC with steel fibers exhibits an apparent percolation threshold of the electrical conductivity, as shown in Figure 12b. With the increase in the MWCNT content from 0.35% to 0.40%, the electrical conductivity (σ_com_) of the HPFRCC increases from 0.018 to 0.10 S/cm and it increases to 0.111 S/cm at 0.50% MWCNT content. The electrical conductivity (σ_rm_) of the MWCNT-reinforced matrix of the 180 MPa HPFRCC increases from 0.0088 to 0.084 S/cm. Figure 12c shows the effect of steel fiber content on the electrical conductivity (σ_rm_) of the unreinforced matrix of the 100 MPa HPFRCC. As the steel fiber content increases from 0% to 1.0%, the electrical conductivity (σ_com_ and σ_m_) increases significantly. As the steel fiber content is increased from 1.0% to 3.0%, the value slightly increases. This indicates that the steel fibers are best dispersed at 1.0% in the 180 MPa HPFRCC and are poorly dispersed above 2.0%, exhibiting fiber clumping and aggregation.

### 3.4. Mercury Intrusion Porosimetry (MIP)

**Figure 13:**
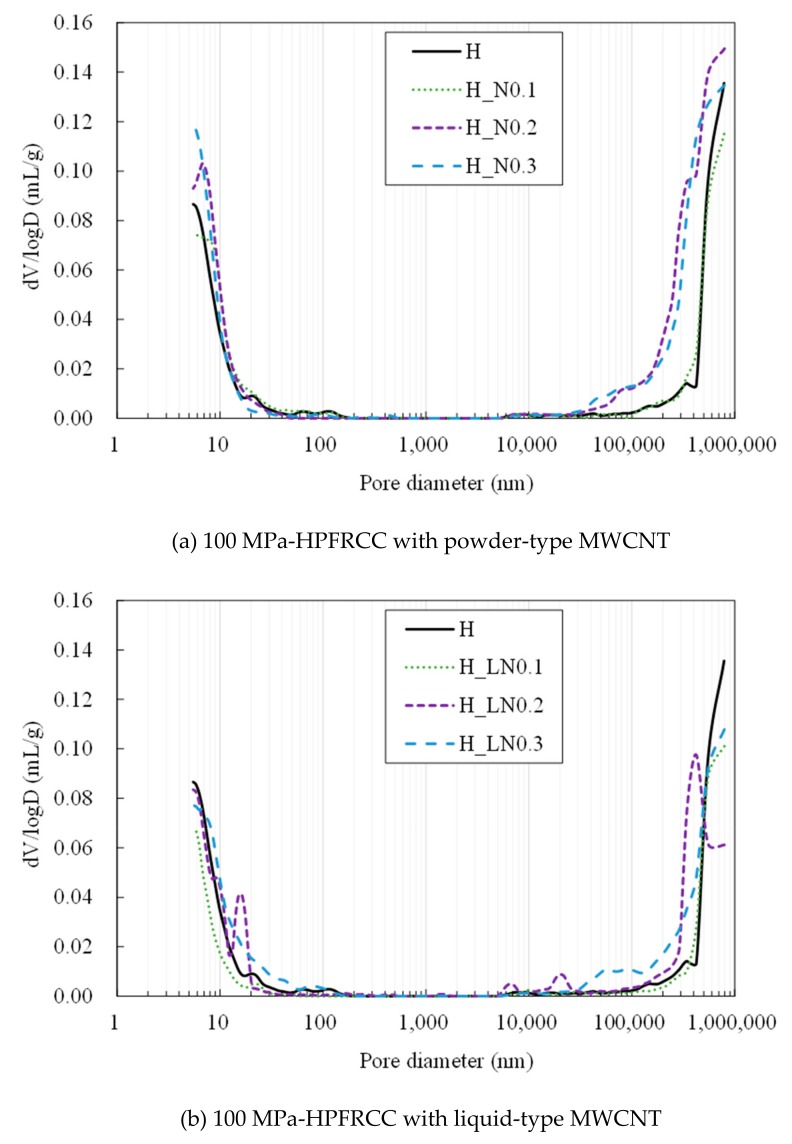

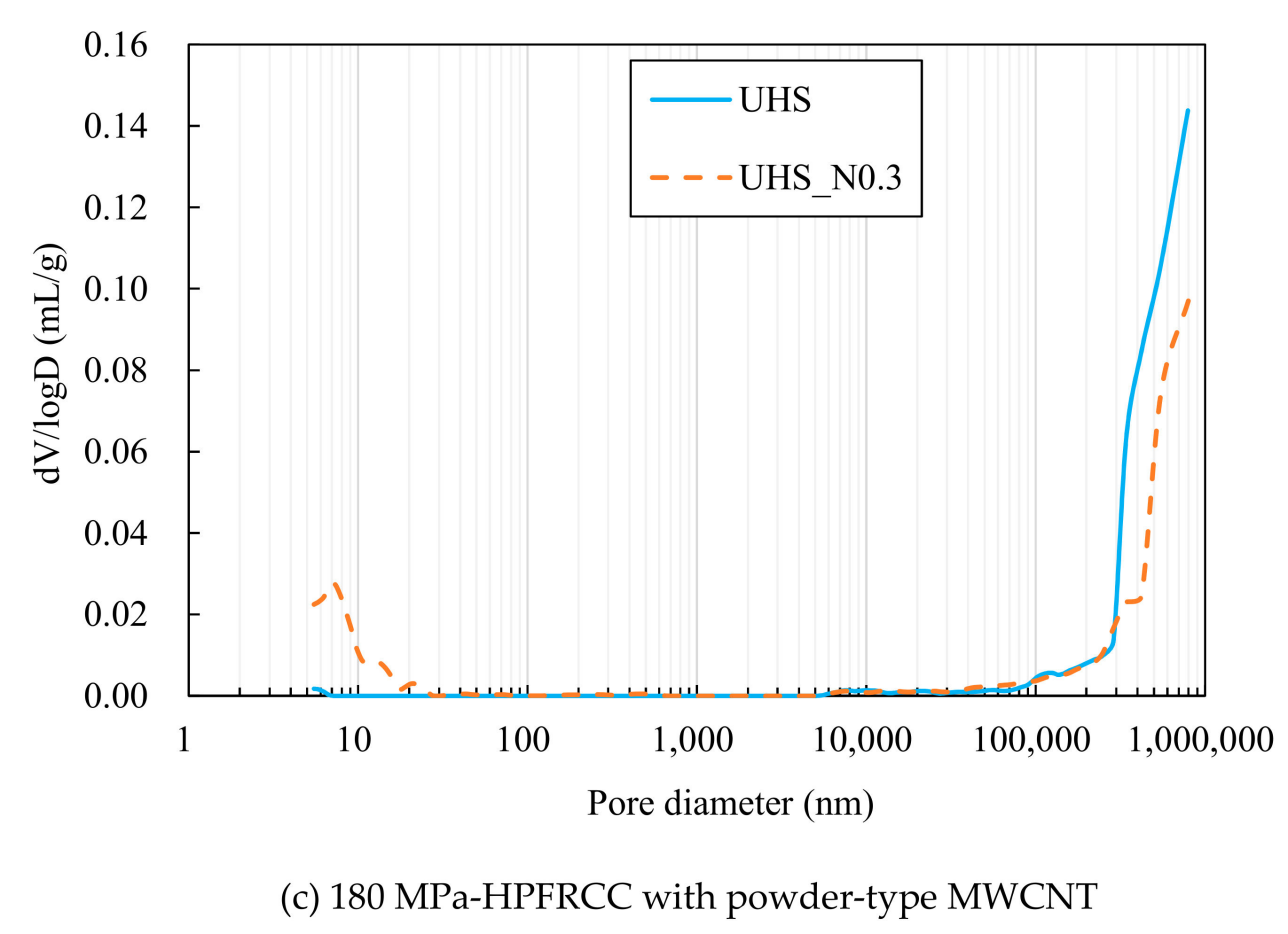
Pore size distributions of 100 MPa and 180 MPa-HPFRCC without steel fiber measured by MIP. (**a**) 100 MPa-HPFRCC with powder-type MWCNT; (**b**) 100 MPa-HPFRCC with liquid-type MWCNT; (**c**) 180 MPa-HPFRCC with powder-type MWCNT.

Figure 13 shows the pore size distribution of the HPFRCC measured using MIP. As listed in Table 5, the volume of the pores with a diameter below 100 nm does not vary regardless of the amount of MWCNTs, whereas the volume of the pores with a diameter above 100 μm increases with the amount of MWCNTs. The increased pore volume is strongly associated with the flowability of the fresh HPFRCC. The flowability decreases with the increase in the MWCNT content, as listed in Table 4. The reduced flowability leads to a poor self-filling property of the HPFRCC, consequently leading to a higher volume of macro pores. Similarly, the use of liquid-type MWCNTs resulted in a higher volume of pores with a diameter of 100 μm because of the reduced flowability. For the 180 MPa HPFRCC, the incorporation of MWCNTs did not decrease the flowability, as the w/b ratio of the 180 MPa HPFRCC with MWCNTs is 0.30, which is higher than that (0.20) of plain HPFRCC without MWCNTs.

### 3.5. Electromagnetic Shielding Effectiveness

**Figure 14:**
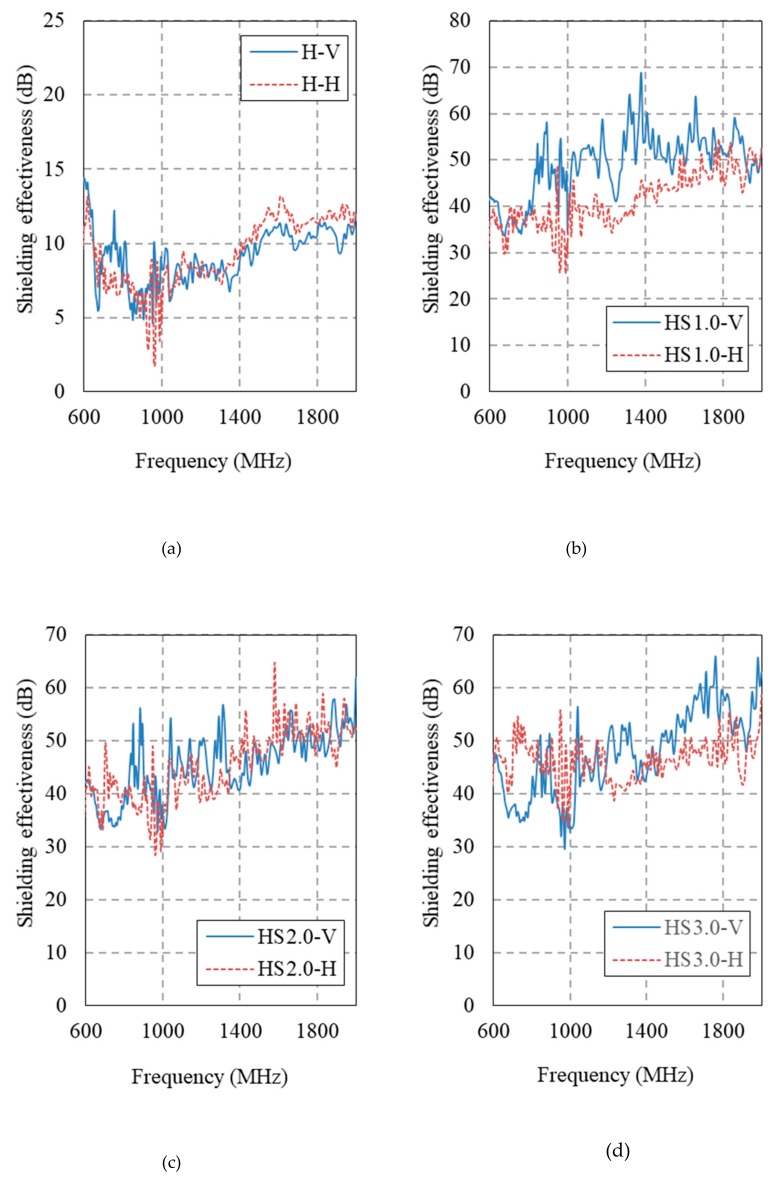

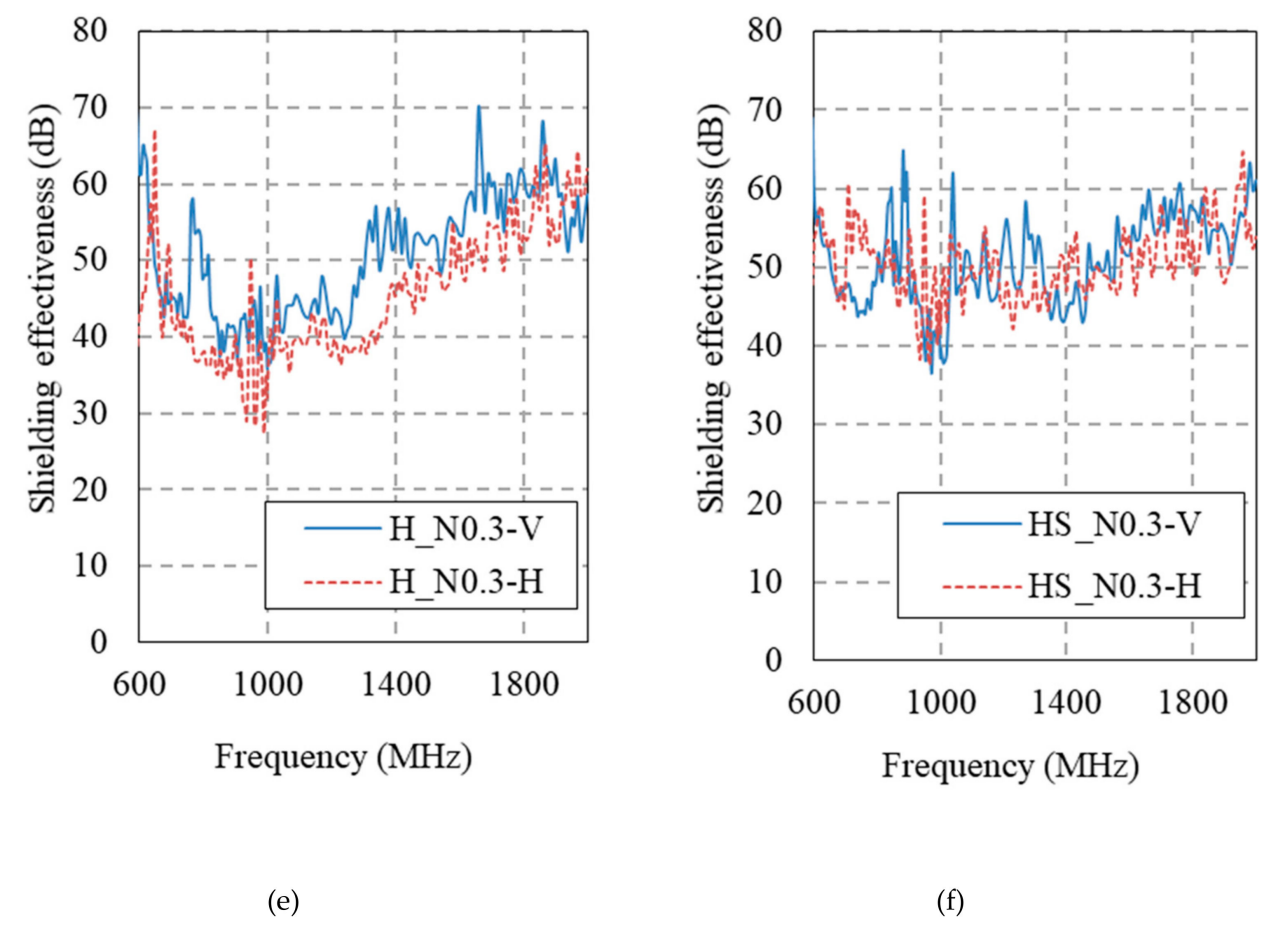
Electromagnetic shielding effectiveness of 100 MPa-HPFRCC. (**a**) Plain; (**b**) 1.0% steel fiber; (**c**) 2.0% steel fiber; (**d**) 3.0% steel fiber; (**e**) 0.3% MWCNT; (**f**) 2.0% steel fiber and 0.3% MWCNT.

**Figure 15:**
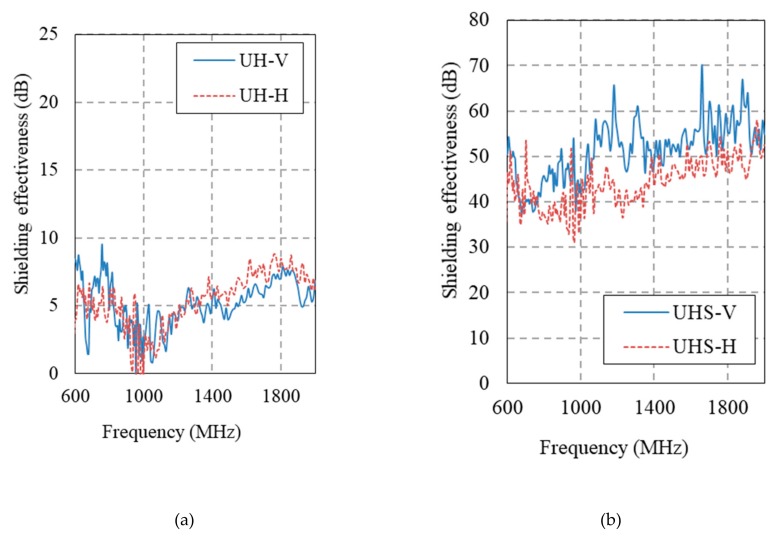

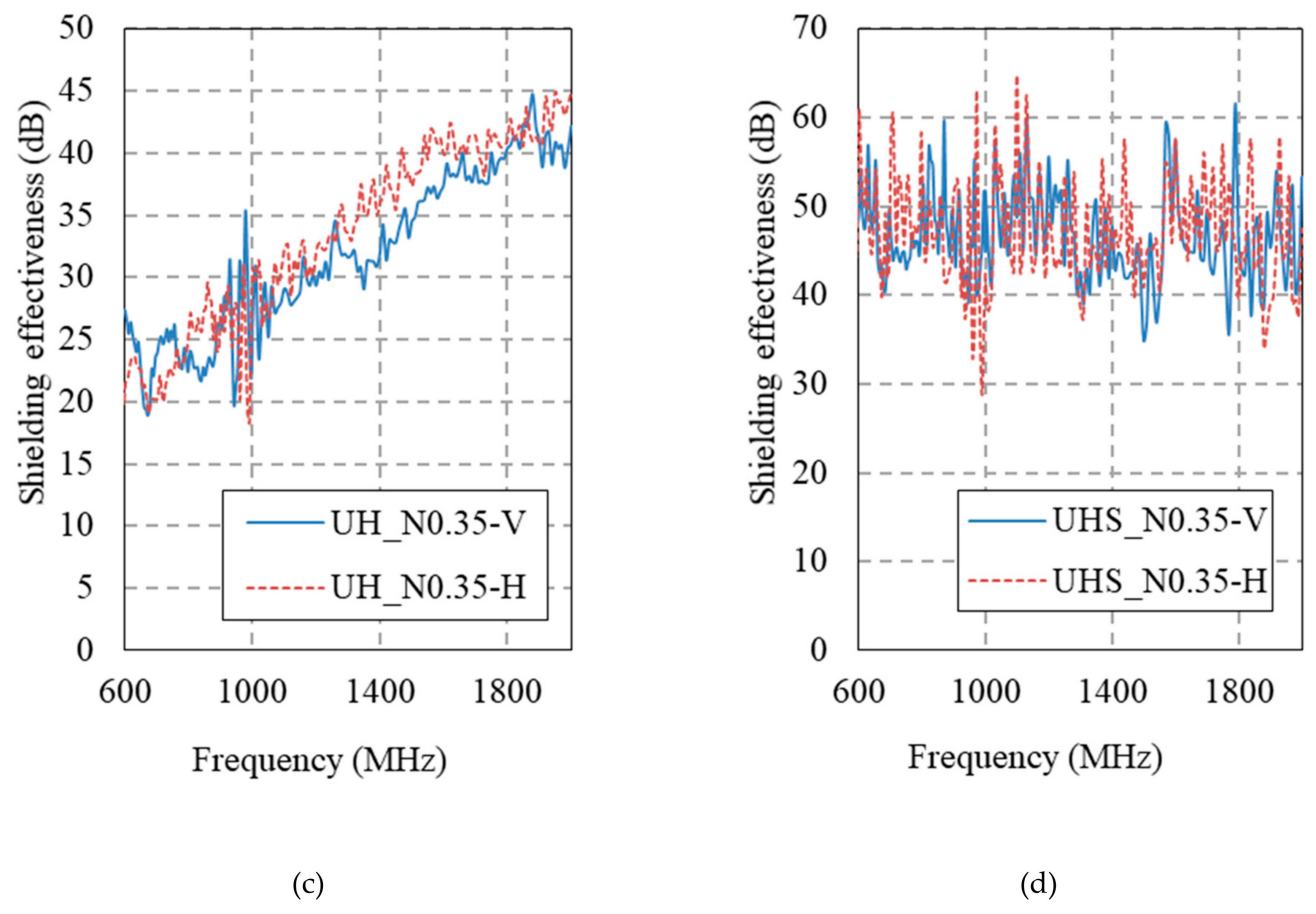
Electromagnetic shielding effectiveness of 180 MPa-HPFRCC. (**a**) Plain; (**b**) 2.0 % steel fiber; (**c**) 0.35% MWCNT; (**d**) 2.0% steel fiber and 0.35% MWCNT.

Figure 14 and Figure 15 show the electromagnetic shielding effectiveness of the HPFRCC measured by conducting an SE test in accordance with MIL-STD-188-125. Table 6 gives a summary of the SE results at 1 GHz.

The SE of the 100 MPa HPFRCC without steel fibers is 6.5 dB at the horizontal antenna and 9.1 dB at the vertical antenna. The SE values of the 180 MPa HPFRCC without steel fibers are 1.4 dB (horizontal) and 2.7 dB (vertical). As the steel fiber content is increased from 0% to 1.0%, the SE increases, as shown in Figure 14a–d. However, the SE does not increase further, even when the steel fiber content is increased from 1.0% to 3.0%. This indicates that the dispersion level of the steel fibers in the HPFRCC is high enough at 1.0%; i.e., the steel fibers in the HPFRCC are well dispersed at 1.0 vol.%. When the fiber content is more than 2.0%, the clumping or aggregation of the steel fibers may occur in the HPFRCC, making it difficult to further increase the SE of the HPFRCC. 

The addition of 0.3% MWCNT leads to a significant increase in the SE of the HPFRCC without steel fibers. The SE increase at 0.3% MWCNT content is as much as that at a steel fiber content of 2.0%. However, the use of both MWCNTs and steel fibers does not increase the SE value as high as expected. At 2.0% steel fiber and 0.3% MWCNT contents, the SE values of the HPFRCC are 45.8 dB (horizontal) and 42.1 dB (vertical), which are slightly higher than those (37.9 dB (horizontal)) from 2.0% steel fiber content and (39.2 dB (horizontal)) at 0.3% MWCNT content. The synergy effect with the use of both steel fibers and MWCNTs was not observed in the HPFRCC. The same can be observed in the SE results of the 180 MPa HPFRCC. 

**Figure 16:**
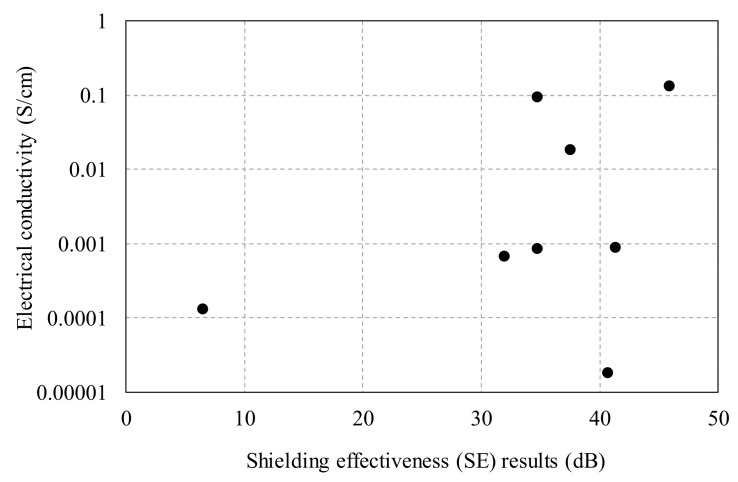
Electromagnetic shielding effectiveness versus electrical conductivity of HPFRCC.

Figure 16 shows that there is no direct relationship between the electrical conductivity and the SE results of the HPFRCC. In particular, the use of steel fibers could not provide any meaningful information between the electrical conductivity and the SE results. At the micro level, the incorporation of steel fibers did not result in an electrical percolation path in the HPFRCC (See Figure 11c); therefore, the electrical conductivity could not be increased. At the macro level, the proper dispersion of the steel fibers into the HPFRCC helped reflect and absorb the electromagnetic waves, increasing the electromagnetic SE. To emphasize the findings once again, although there was no electrical percolation path in the HPFRCC, a high electromagnetic SE could be achieved. The electrical conductivity of the HPFRCC with 2.0% steel fiber content was 0.00086 S/cm, and the SE values were 45.8 dB (horizontal) and 42.1 dB (vertical) at 1 GHz. Despite the electrical conductivity of the HPFRCC being as low as 10^−5^ S/cm, the SE value was as high as 40 dB. The formation of the electrical percolation path with the addition of MWCNTs had a positive effect on the increase in the electromagnetic SE. The electrical conductivity of the HPFRCC with 0.3% MWCNT content was 0.093 S/cm, which was high enough to form a percolation path, and the SE values were 34.7 dB (horizontal) and 39.2 dB (vertical) at 1 GHz. 

In summary, the incorporation of steel fibers at the macro level can help improve the electromagnetic SE regardless of the formation of percolation paths, which is strongly associated with the electrical conductivity, whereas the incorporation of MWCNTs at the micro level can help improve the electromagnetic SE only when percolation paths are formed in the cement matrix. To support these findings, further study needs to be conducted.

## 4. Concluding Remarks

This study presented experimental results and discussions pertaining to the electrical conductivity and electromagnetic shielding effectiveness of HPFRCCs. To improve the electrical conductivity of HPFRCCs, MWCNTs and steel fibers were added to the HPFRCC. The electrical conductivity was measured using the AC-IS method, and the electromagnetic shielding effectiveness was measured inside a dual shielding room in accordance with MIL-STD-188-125. The following conclusions can be drawn from the results presented in this paper.

1. The electrical conductivity of the 100 MPa HPFRCC with 0.30% MWCNT was 0.093 S/cm and that of the 180 MPa HPFRCC with 0.4% MWCNT and 2.0% steel fiber was 0.10 S/cm. To achieve a high electrical conductivity, adding MWCNTs was more beneficial than adding steel fibers.
2. At 2.0% steel fiber and 0.3% MWCNT contents, the SE values of the HPFRCC were found to be 45.8 dB (horizontal) and 42.1 dB (vertical), which were slightly higher than those (37.9 dB (horizontal)) at 2.0% steel fiber content and that (39.2 dB (horizontal)) at 0.3% MWCNT content.
3. The synergy effect in the use of both steel fibers and MWCNTs on the shielding effectiveness was not observed in the HPFRCC. There was no direct relationship between the electrical conductivity and the SE results of the HPFRCC with MWCNTs and steel fibers. The incorporation of steel fibers did not result in any electrical percolation path in the HPFRCC at the micro level; therefore, a high electrical conductivity could not be achieved. At the macro level, the proper dispersion of the steel fibers into the HPFRCC helped reflect and absorb the electromagnetic waves, consequently increasing the electromagnetic SE. Although there was no electrical percolation path in the HPFRCC, a high electromagnetic SE could be achieved.
4. The electrical conductivity of the HPFRCC with 2.0% steel fiber content was 0.00086 S/cm, and the SE values were 45.8 dB (horizontal) and 42.1 dB (vertical) at 1 GHz. Despite the electrical conductivity of the HPFRCC being as low as 10^−5^ S/cm, the SE value was as high as 40 dB. The formation of the electrical percolation path with the addition of MWCNTs had a positive effect on the electromagnetic SE. The electrical conductivity of the HPFRCC with 0.3% MWCNT content was 0.093 S/cm, which was high enough to form a percolation path, and the SE values were 34.7 dB (horizontal) and 39.2 dB (vertical) at 1 GHz.
5. In summary, the incorporation of steel fibers at the macro level can help improve the electromagnetic SE regardless of the formation of percolation paths, whereas the incorporation of MWCNTs at the micro level can help improve the electromagnetic SE only when percolation paths are formed in the cement matrix.

## Figures and Tables

**Table 1:** Chemical composition of binder materials used in this study.

(wt %)	XRF			Rietveld Analysis	
	OPC	Fly Ash	Silica Fume	Compound	OPC
SiO_2_	20.6	38.07	95.31	C_3_S	62.2
Al_2_O_3_	5.0	14.54	0.1	C_2_S	11.0
Fe_2_O_3_	3.4	5.42	0.35	C_4_AF	11.0
CaO	60.7	22.78	0.21	C_3_A	4.2
MgO	2.6	2.67	0.8	Calcite	5.1
SO_3_	2.38	5.45	0.55	Gypsum	0.7
K_2_O	0.98	5.83	-	Anhydrite	-
Na_2_O	0.15	0.92	0.19	Ye’elimite (C)	-
TiO2	0.27	3.62	-	Ye’elimite (O)	-
P2O5	0.11	1.52	0.03	CT	-
Others	<0.25	1.19	-	C2AS	-
LOI	0.75	7.1	2.46	Hemihydrate	-
				Quartz	-
				Arcanite	-
				Others	2.2

**Table 2:** Physical properties of steel fiber adopted for the high-performance, fiber-reinforced cementitious composite (HPFRCC).

TYPE OF FIBER	Density (kg/cm^3^)	Tensile Strength (MPa)	Length (mm)	$l_{f}/d_{f}$ (mm/mm)
Straight	7.8	2500	19.5	97.5

**Table 3:** Mix ratio of HPFRCC.

Mixture	w/b ^a^ Ratio (w/c)	OPC	Fly Ash	Micro Silica	CNT (%)		Fine Aggregate	Fill-er	SP Agent	Steel Fiber ^c^ (%)
					Powder	Liquid ^b^				
H	0.3 (0.35)	1	0.2	0.1	-	-	1.2	0.2	0.015	-
H_N0.1	0.3 (0.35)	1	0.2	0.1	0.1	-	1.2	0.2	0.018	-
H_N0.2	0.3 (0.35)	1	0.2	0.1	0.2	-	1.2	0.2	0.022	-
H_N0.3	0.3 (0.35)	1	0.2	0.1	0.3	-	1.2	0.2	0.026	-
H_N0.4	0.3 (0.35)	1	0.2	0.1	0.4	-	1.2	0.2	-	-
H_NN0.3	0.3 (0.35)	1	0.2	0.1	0.3	-	1.2	0.2	0.026	-
H_LN0.1	0.3 (0.35)	1	0.2	0.1	-	0.1	1.2	0.2	0.018	-
H_LN0.2	0.3 (0.35)	1	0.2	0.1	-	0.2	1.2	0.2	0.022	-
H_LN0.3	0.3 (0.35)	1	0.2	0.1	-	0.3	1.2	0.2	0.026	-
HS	0.3 (0.35)	1	0.2	0.1	-	-	1.2	0.2	0.015	2.0
HS_N	0.3 (0.35)	1	0.2	0.1	0.3	-	1.2	0.2	0.030	2.0
HS_LN	0.3 (0.35)	1	0.2	0.1	-	0.3	1.2	0.2	0.030	2.0
UHS	0.2 (0.25)	1	-	0.25	-	-	1.2	0.2	0.051	2.0
UHS_LN0.3w	0.25 (0.30)	1	-	0.25	0.3	-	1.2	0.2	0.066	2.0
UHS_LN0.3	0.3 (0.35)	1	-	0.25	0.3	-	1.2	0.2	0.032	2.0
UHS_N0.35	0.3 (0.35)	1	-	0.25	0.35	-	1.2	0.2	0.032	2.0
UHS_N0.4	0.3 (0.35)	1	-	0.25	0.4	-	1.2	0.2	0.032	2.0
UHS_N0.5	0.3 (0.35)	1	-	0.25	0.5	-	1.2	0.2	0.035	2.0
UHS_LN0.3	0.3 (0.35)	1	-	0.25	-	0.3	1.2	0.2	0.026	2.0
UHS_LN0.5	0.3 (0.35)	1	-	0.25	-	0.5	1.2	0.2	0.026	2.0

^a^ Binder is a sum of cement, microsilica and fly ash. ^b^ Solid MWCNT content in MWCNT liquid solution is expressed as a percentage (%) by wt. of cement. ^c^ It indicates a volume fraction of steel fibers in HPFRCC.

**Table 4:** Slump flow results.

Mixture (without Steel Fiber)	Mini Slump Flow (mm)	Mixture (with Steel Fiber)	Mini Slump Flow (mm)
H	200	H100	240
N0.1	215	H100N	130
N0.2	150	H100SN	160
N0.3	150	H180	250
N0.4	-	H180N0.3w	150
NN0.3	160	H180N0.3	230
SN0.1	250	H180N0.35	170
SN0.2	220	H180N0.4	160
SN0.3	170	H180N0.5	150
		H180SN0.3	200
		H180SN0.5	160

**Table 5:** Pore characteristics of HPFRCC as measured by MIP.

Sample	Pore Volume (mL/g)		Porosity (%)	Total Intrusion Volume (mL/g)
	5–100 nm	1–1000 μm		
H	0.031	0.043	15.83	0.0819
H_N0.1	0.028	0.030	11.36	0.0582
H_N0.2	0.031	0.070	18.14	0.1012
H_N0.3	0.028	0.065	16.62	0.0933
H_LN0.1	0.014	0.027	8.39	0.042
H_LN0.2	0.026	0.037	11.97	0.064
H_LN0.3	0.031	0.042	14.24	0.074
UHS	<0.001	0.045	9.12	0.046
UHS_N0.3	0.008	0.029	7.37	0.037

**Table 6:** SE results at 1 GHz.

Sample	Frequency (MHz)	Horizontal (dB)	Vertical (dB)
H	1000	6.5	9.06
HS1.0	1000	31.86	47.33
HS2.0	1000	34.65	37.9
HS3.0	1000	41.25	35.59
H_N0.3	1000	34.72	39.19
HS_N0.3	1000	45.82	42.11
UH	1000	1.42	2.72
UHS	1000	40.65	43.91
UH_N0.35	1000	28.37	21.87
UHS_N0.35	1000	37.47	51.72
